# Advancements and challenges in personalized neoantigen-based cancer vaccines

**DOI:** 10.3389/or.2025.1541326

**Published:** 2025-03-14

**Authors:** Parminder Singh, Mahalaqua Nazli Khatib, Roopashree R, Mandeep Kaur, Manish Srivastava, Amit Barwal, G. V. Siva Rajput, Pranchal Rajput, Rukshar Syed, Gajendra Sharma, Sunil Kumar, Muhammed Shabil, Sakshi Pandey, Manvinder Brar, Ganesh Bushi, Rachana Mehta, Sanjit Sah, Khang Wen Goh, Prakasini Satapathy, Abhay M. Gaidhane, Shailesh Kumar Samal

**Affiliations:** ^1^ Center for Global Health Research, Saveetha Medical College and Hospital, Saveetha Institute of Medical and Technical Sciences, Saveetha University, Chennai, India; ^2^ Faculty of Data Science and Information Technology, INTI International University, Nilai, Malaysia; ^3^ Division of Evidence Synthesis, Global Consortium of Public Health and Research, Datta Meghe Institute of Higher Education, Wardha, India; ^4^ Department of Chemistry and Biochemistry, School of Sciences, JAIN (Deemed to be University), Bangalore, Karnataka, India; ^5^ Department of Allied Healthcare and Sciences, Vivekananda Global University, Jaipur, Rajasthan, India; ^6^ Department of Endocrinology, NIMS University, Jaipur, India; ^7^ Chandigarh Pharmacy College, Chandigarh Group of College, Mohali, Punjab, India; ^8^ Department of Chemistry, Raghu Engineering College, Visakhapatnam, Andhra Pradesh, India; ^9^ School of Applied and Life Sciences, Division of Research and Innovation, Uttaranchal University, Dehradun, India; ^10^ IES Institute of Pharmacy, IES University, Bhopal, Madhya Pradesh, India; ^11^ New Delhi Institute of Management, Tughlakabad Institutional Area, New Delhi, India; ^12^ Department of Microbiology, Graphic Era (Deemed to be University), Dehradun, India; ^13^ Noida Institute of Engineering and Technology (Pharmacy Institute), Greater Noida, India; ^14^ Centre of Research Impact and Outcome, Chitkara University, Rajpura, Punjab, India; ^15^ Chitkara Centre for Research and Development, Chitkara University, Solan, Himachal Pradesh, India; ^16^ School of Pharmaceutical Sciences, Lovely Professional University, Phagwara, India; ^17^ Clinical Microbiology, RDC, Manav Rachna International Institute of Research and Studies, Faridabad, Haryana, India; ^18^ Department of Paediatrics, Dr. D. Y. Patil Medical College Hospital and Research Centre, Dr. D. Y. Patil Vidyapeeth (Deemed-to-be-University), Pimpri, Pune, Maharashtra, India; ^19^ Department of Public Health Dentistry, Dr. D. Y. Patil Medical College Hospital and Research Centre, Dr. D. Y. Patil Vidyapeeth (Deemed-to-be-University), Pimpri, Pune, Maharashtra, India; ^20^ Department of Medicine, Korea Universtiy, Seoul, Republic of Korea; ^21^ Faculty of Mathematics and Natural Sciences, Universitas Negeri Padang, Padang, Indonesia; ^22^ University Center for Research and Development, Chandigarh University, Mohali, Punjab, India; ^23^ Medical Laboratories Techniques Department, AL-Mustaqbal University, Hillah, Babil, Iraq; ^24^ Jawaharlal Nehru Medical College, and Global Health Academy, School of Epidemiology and Public Health, Datta Meghe Institute of Higher Education, Wardha, India; ^25^ Unit of Immunology and Chronic Disease, Institute of Environmental Medicine, Karolinska Institutet, Stockholm, Sweden

**Keywords:** neoantigen-based vaccines, cancer immunotherapy, personalized medicine, vaccine delivery systems, immunogenicity prediction

## Abstract

Advancements in personalized neoantigen-based cancer vaccines are ushering in a new era in oncology, targeting unique genetic alterations within tumors to enhance treatment precision and efficacy. Neoantigens, specific to cancer cells and absent in normal tissues, are at the heart of these vaccines, promising to direct the immune system specifically against the tumor, thereby maximizing therapeutic efficacy while minimizing side effects. The identification of neoantigens through genomic and proteomic technologies is central to developing these vaccines, allowing for the precise mapping of a tumor’s mutational landscape. Despite advancements, accurately predicting which neoantigens will elicit strong immune responses remains challenging due to tumor variability and the complexity of immune system interactions. This necessitates further refinement of bioinformatics tools and predictive models. Moreover, the efficacy of these vaccines heavily depends on innovative delivery methods that enhance neoantigen presentation to the immune system. Techniques like encapsulating neoantigens in lipid nanoparticles and using viral vectors are critical for improving vaccine stability and delivery. Additionally, these vaccines contribute towards achieving Sustainable Development Goal 3.8, promoting universal health coverage by advancing access to safe and effective cancer treatments. This review delves into the potential of neoantigen-based vaccines to transform cancer treatment, examining both revolutionary advancements and the ongoing challenges they face.

## Introduction

The development of personalized neoantigen-based cancer vaccines represents a significant breakthrough in oncology, heralding a transformative shift towards precision medicine (1). These vaccines target specific mutations unique to an individual’s tumor, known as neoantigens, which are not present in normal tissues (2, 3). This approach promises enhanced treatment efficacy and minimized adverse effects by directing the immune system to specifically target and destroy only the cancer cells exhibiting these unique antigens. The specificity of neoantigen-based vaccines utilizes the body’s natural immune responses, potentially revolutionizing cancer treatment by making therapies more targeted and safer for healthy cells (4).

The process of developing these vaccines is highly sophisticated, relying on the detailed identification and selection of neoantigens through comprehensive genomic and proteomic analyses. Advances in sequencing technology allow researchers to map the entire mutational landscape of individual tumors. This mapping helps identify unique mutations that can be targeted by vaccines (5). However, a major challenge in this process is the accurate prediction of which neoantigens will effectively stimulate a robust immune response. Not all mutations result in neoantigens capable of triggering the necessary T-cell response to attack the tumor, making the selection process crucial for the vaccine’s success (6). Bioinformatics tools are integral to this selection, utilizing algorithms to predict the binding affinity of potential neoantigen peptides to Major Histocompatibility Complex (MHC) molecules—a key step in determining their immunogenicity (7). Despite technological advances, these predictive models need ongoing refinement to enhance their accuracy and reliability, as the vaccine’s success heavily depends on the chosen neoantigens’ immunogenicity.

Furthermore, the success of these vaccines also hinges on the delivery systems used to administer these vaccines play a critical role in their overall success. Effective delivery ensures that neoantigens are presented in a manner that optimally stimulates the immune system. Recent innovations in this area include the development of sophisticated delivery mechanisms, such as lipid nanoparticles and viral vectors, designed to enhance the stability and cellular uptake of neoantigens (8). These systems aim to improve the immunogenic presentation of neoantigens, thereby enhancing the body’s immune response against the tumor. The challenge lies in achieving targeted delivery and controlled release of neoantigens, which are critical for initiating and maintaining an effective anti-tumor immune response (9). Moreover, the production processes for these personalized vaccines are complex and must be both cost-effective and scalable to ensure they can be widely used in clinical settings without prohibitive expenses.

This review includes detailed discussions on various topics such as source of neoantigens, advancements in vaccine delivery systems, and the challenges of integrating these vaccines with other therapeutic strategies. The development of personalized neoantigen-based cancer vaccines also aligns with global health objectives, notably Sustainable Development Goal 3.8, which aims to achieve universal health coverage (10). This includes access to quality essential healthcare services and safe, effective, and affordable essential medicines and vaccines for all. By enhancing the precision and affordability of cancer treatments through tailored vaccine therapies, this approach contributes significantly towards reducing health inequities and improving access to life-saving treatments worldwide.

## Background

**FIGURE 1:**
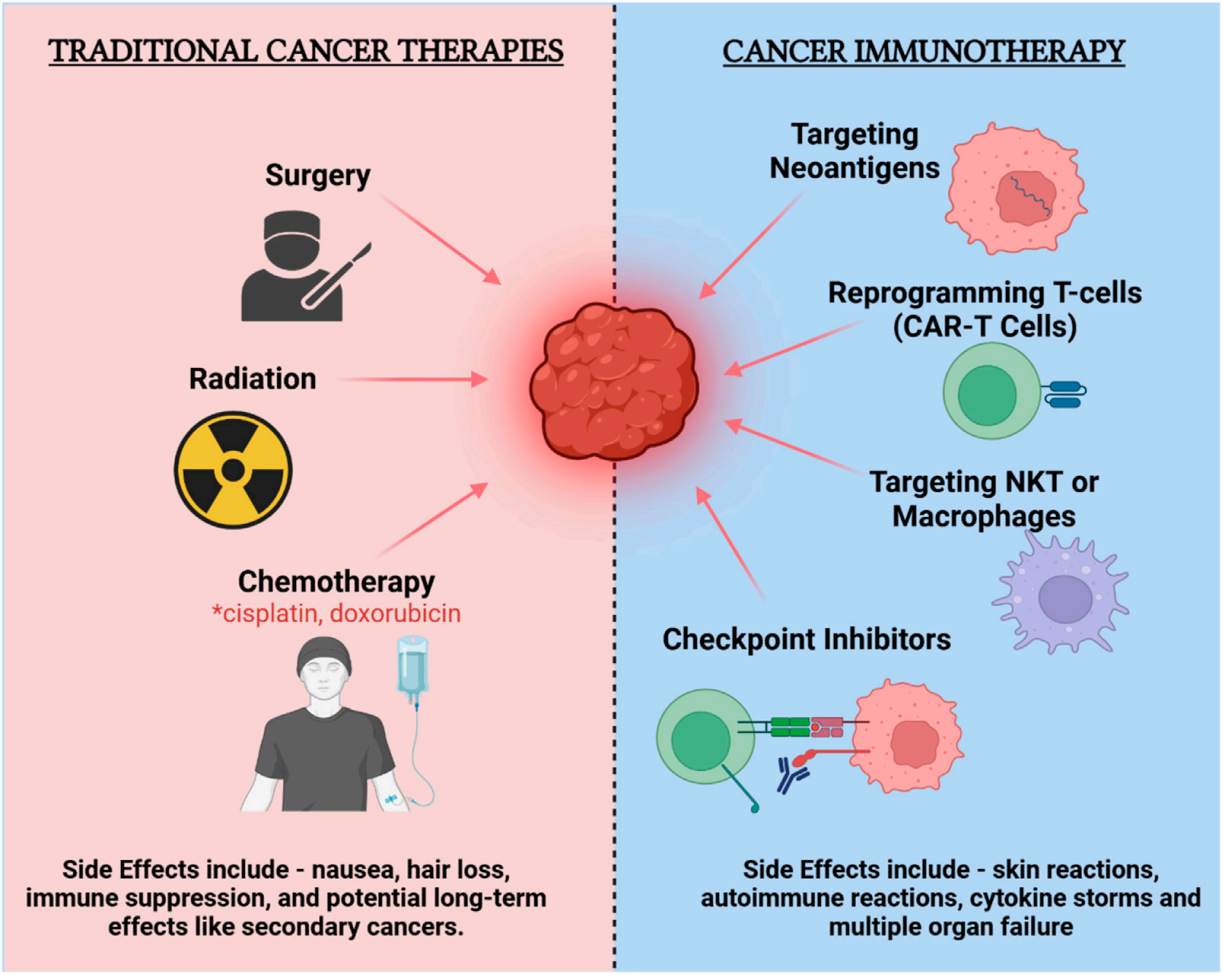
Comparison of traditional cancer therapies and cancer immunotherapy: efficacy and side effects.

Cancer immunotherapy has emerged as a transformative approach in oncology, fundamentally reshaping how various malignancies are treated (4, 11). The field has evolved significantly, from the early use of non-specific immune stimulants to the latest generation of targeted therapies, including immune checkpoint inhibitors, which have demonstrated remarkable success in activating the immune system against cancer cells (Figure 1). These developments have greatly benefited from the identification of cancer-specific biomarkers, enhancing the precision of oncological interventions (12). Parallel to these advancements, the concept of neoantigens has garnered attention. Neoantigens are tumor-specific antigens that arise due to mutations unique to each tumor, making them ideal targets for personalized immunotherapies. This specificity is critical as it allows for the targeting of cancer cells while minimizing impact on normal tissues, thereby enhancing treatment efficacy and reducing adverse effects (13).

The rationale for personalized vaccines targeting neoantigens is rooted in their ability to provoke a robust immune response specifically against cancer cells, bypassing the limitations of traditional therapies which often target broader cell populations, leading to significant side effects and variable efficacy (14). Personalized neoantigen vaccines leverage the precision of immune targeting to improve the specificity of cancer immunotherapy, offering a promising avenue for enhancing patient outcomes in oncology (15). However, the implementation of these vaccines faces challenges, including the complexity of accurately identifying and predicting the immunogenicity of neoantigens, which remains a critical area of ongoing research and development. The integration of these vaccines into clinical practice holds the potential to significantly improve the precision and effectiveness of cancer treatment, marking a critical step forward in the evolution of cancer immunotherapy.

### Molecular sources of neoantigens

**FIGURE 2:**
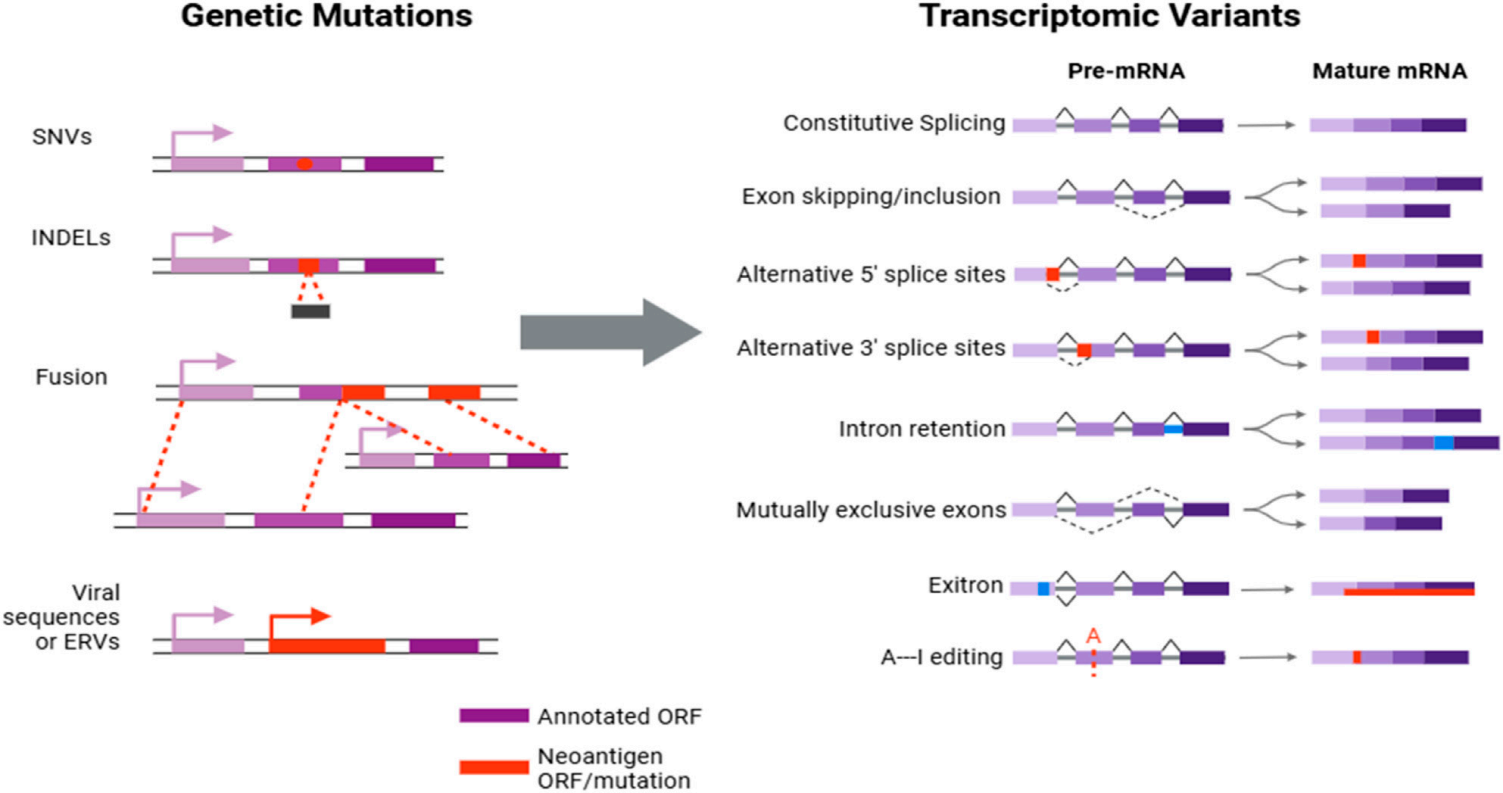
Transcriptomic mechanisms leading to neoantigen production in cancer.

The sources of cancer neoantigens are diverse, encompassing a wide range of genomic alterations that contribute to the immunogenic landscape of tumors (Figure 2). Traditional neoantigens arise from single-nucleotide variants (SNVs) and small insertion/deletion mutations (indels), which directly alter amino acid sequences and create novel protein fragments that can be recognized by the immune system (16). However, contemporary research has illuminated additional mechanisms through which neoantigens can be generated, thereby expanding the potential targets for immunotherapeutic interventions.

Alternative splicing events modify RNA transcripts to produce variant proteins with sequences distinct from the canonical forms. These splice variants often introduce unique epitopes into the tumor cell’s presentation repertoire, significantly enhancing its visibility to immune surveillance (17). Gene fusions, resulting from chromosomal rearrangements, also contribute to the neoantigen pool by creating chimeric proteins that combine sequences from different genes. These chimeric junctions form novel peptide sequences that are highly immunogenic because they are not present in normal cells (18). Post-translational modifications, such as glycosylation or phosphorylation, can further alter the epitope landscape of tumor cells by modifying peptide presentation and recognition by T cells (16). Moreover, the reactivation of endogenous retroviral elements, often silenced in normal cells but activated in cancer cells due to epigenetic changes, introduces additional unique peptides that can be processed and presented as neoantigens (19). Importantly, the integration of viral DNA into the host genome in virus-associated cancers can also generate viral-derived tumor antigens. These antigens are particularly compelling targets for immunotherapy because they are foreign to the human immune system, enhancing the likelihood of a robust immune response (20). Viruses such as human papillomavirus in cervical cancer and Epstein-Barr virus in nasopharyngeal carcinoma are known to contribute to oncogenesis through such mechanisms.

The identification and validation of these diverse sources of neoantigens are supported by advanced computational tools and databases, which predict the immunogenic potential of these complex genomic alterations. Tools like NeoSplice and databases like MONET facilitate the exploration of neoantigens arising from non-standard genetic events, including those induced by viral integration (21, 22). These developments are pivotal in broadening the scope of neoantigen discovery and enhancing the efficacy of personalized cancer vaccines, leading to more effective cancer immunotherapies that leverage the full spectrum of neoantigen diversity.

### Mechanisms of personalised neoantigen-based cancer vaccines

**FIGURE 3:**
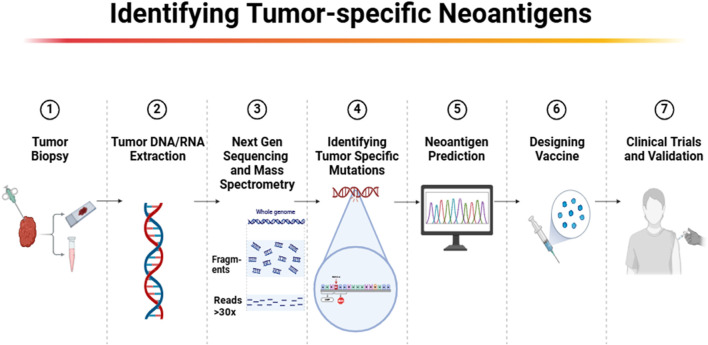
Workflow for identifying tumor-specific neoantigens for personalized cancer immunotherapy.

The process of identifying tumor-specific mutations for personalized neoantigen-based cancer vaccines begins with cutting-edge sequencing technologies such as whole exome sequencing and RNA sequencing (Figure 3). These technologies enable the precise identification of mutations unique to each patient’s tumor, providing a basis for targeting these mutations with customized vaccines (23). These identified mutations, absent in normal tissues but expressed in tumor cells, represent potential targets for vaccine development because they are likely to be recognized as foreign by the immune system (14). By focusing on neoantigens that arise from these unique mutations, these vaccines can be tailored to each individual’s cancer, enhancing the specificity and effectiveness of the treatment. Recent advancements have improved our ability to predict and select highly immunogenic neoantigens. Tools such as the HANSolo algorithm and the NeoSplice system utilize complex bioinformatics algorithms that assess mutation frequency, peptide binding affinity to MHC molecules, and the immunogenic potential of peptide sequences derived from tumor RNA transcripts (21, 24). However, despite the advancements, current bioinformatics tools face significant limitations. One major challenge is the predictive accuracy of these algorithms, which often cannot account for the full complexity of immune responses. For example, peptide-MHC binding predictions may not always align with actual *in vivo* immunogenicity, as these tools primarily rely on binding affinity models that do not capture all immune system interactions, such as the influence of co-stimulatory molecules or immune checkpoint inhibitors (25). Furthermore, the predictive power of algorithms is also limited by the availability and quality of reference data for different populations, which may not adequately represent the genetic diversity found in patients globally.

**FIGURE 4:**
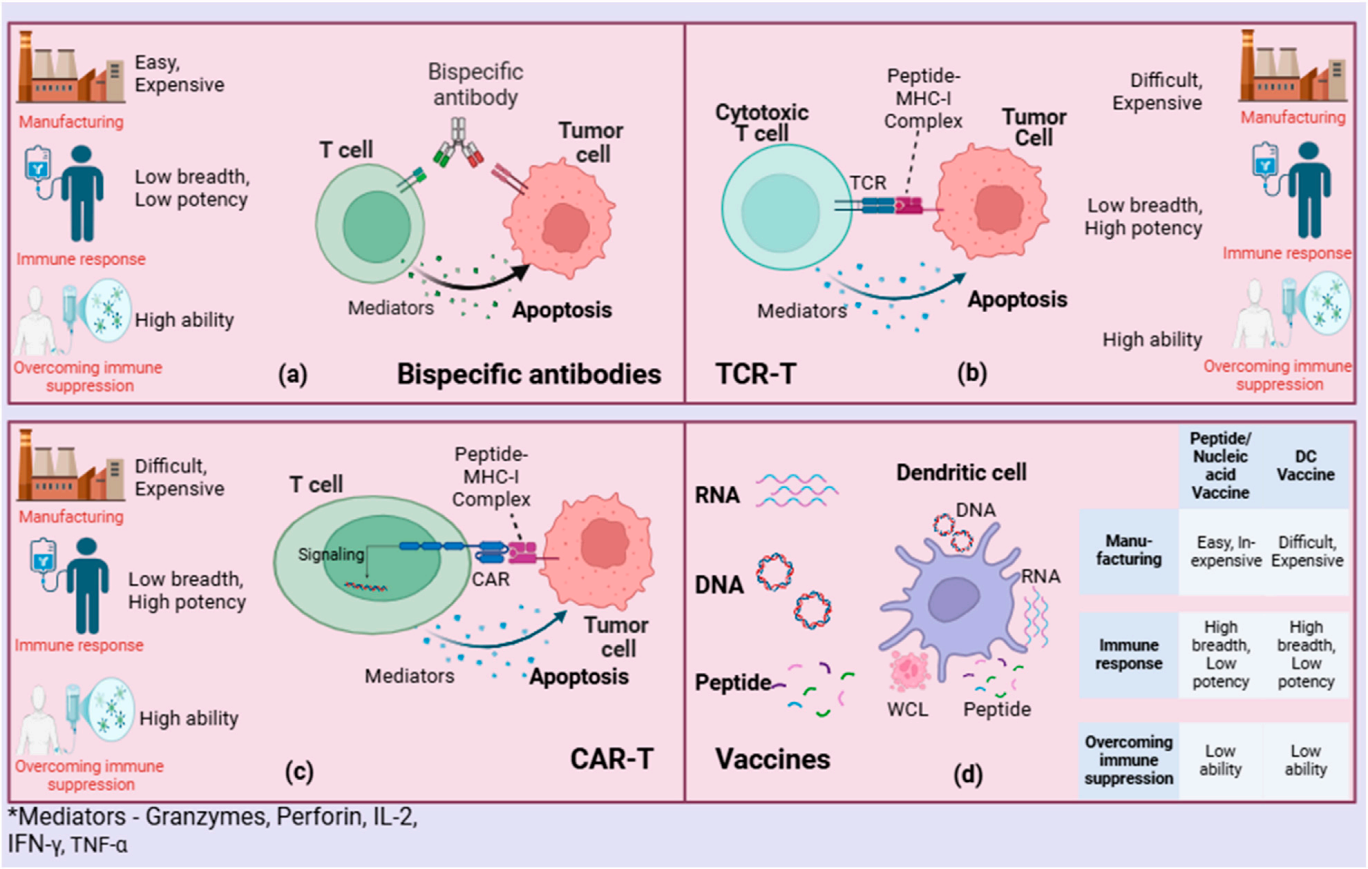
Comparing neoantigen-based therapies: **(A)** Bispecific antibodies, **(B)** TCR-T, **(C)** CAR-T, and **(D)** Vaccines.

Additionally, while these bioinformatics tools have shown some success in predicting potential neoantigens, there remains a substantial gap in accurately forecasting which neoantigens will truly provoke a robust immune response. The immunogenicity of neoantigens can be influenced by the subcellular location of source proteins, with studies showing that peptides from certain cellular compartments are more likely to be processed and presented by MHC molecules, enhancing T-cell recognition and activation (26). In the design of these vaccines, various platforms are utilized, including peptide-based, DNA, RNA, and viral vector-based approaches (Figure 4D). Each method has its advantages in how it presents neoantigens to the immune system.

#### Peptide-based vaccines

Peptide-based vaccines involve synthesizing short peptides that match the neoantigen sequences. A notable study involving the peptide-based neoantigen vaccine EVX-01, formulated with the liposomal adjuvant CAF09b, has shown encouraging results in patients with metastatic melanoma. This dose-escalation study, combined with anti-PD-1 therapy, was administered at increasing dosages, showing a strong safety profile and substantial immunogenicity (27). The highest dose level achieved a 100% clinical response rate among treated patients, with 67% of all participants experiencing objective tumor responses, including complete and partial responses. Additionally, several peptide-based vaccines such as NeuVax (Nelipepimut-S), PolyPEPI1018, Montanide, UCPVax, and TG4050 have been developed and tested in clinical trials across various cancer types, enhancing the landscape of cancer immunotherapy. NeuVax, targeting HER2 for breast cancer, and PolyPEPI1018 combined with Montanide for colorectal cancer, have shown particular promise, enhancing T-cell responses and potentially leading to prolonged disease control (28, 29). Similarly, UCPVax has been explored in HPV-positive cancers to boost immune response when combined with checkpoint inhibitors, reflecting a synergistic approach to vaccine therapy (30). TG4050, a personalized cancer vaccine using a viral vector, reported from a phase I trial, demonstrated immunogenicity and safety in treating head and neck carcinoma, underscoring the potential of these vaccines in managing complex cancer profiles (31). Another innovative approach in peptide vaccine development involves the use of dual-modified iron oxide nanoparticles, which target and repolarize tumor-associated macrophages (TAMs) from a pro-tumor M2 to an anti-tumor M1 phenotype (32). This strategy, aimed at overcoming the immunosuppressive tumor microenvironment, significantly enhances the infiltration of CD8 (+) T cells into the tumor and activates dendritic cells in sentinel lymph nodes, leading to inhibited tumor growth and a 40% cure rate in a preclinical model.

#### DNA-based vaccines

DNA-based neoantigen vaccines are gaining prominence in the field of personalized cancer immunotherapy, offering targeted approaches to stimulate the immune system against specific tumor mutations. DNA vaccines consist of plasmid DNA that encodes tumor-specific antigens, which are directly delivered into the host cells to stimulate an immune response (33). This strategy is advantageous as it can elicit both humoral and cellular immunity without the need for live pathogens. One example of a DNA vaccine is the ERBB2 ICD plasmid-based vaccine, which targets the ERBB2 receptor commonly overexpressed in various cancers, including breast and ovarian cancers. This vaccine has demonstrated potential in generating a strong immune response by inducing T-cell activation against the ERBB2 antigen, a promising strategy for improving anti-cancer immunity (34). Another example is GX-188E, a DNA vaccine designed to target the E6 and E7 oncoproteins of human papillomavirus (HPV), a key factor in the development of cervical and other HPV-related cancers. GX-188E has shown promising results in clinical trials, particularly in patients with advanced cervical cancer, by stimulating the immune system to specifically target and destroy HPV-infected cells (35). Similarly, PAP with GM-CSF adjuvant is a DNA vaccine targeting prostatic acid phosphatase (PAP), an antigen commonly found in prostate cancer. The inclusion of GM-CSF as an adjuvant enhances dendritic cell activation and antigen presentation, improving the overall immune response to the PAP antigen (36).

A more innovative approach involves a spleen-targeted neoantigen DNA vaccine designed for hepatocellular carcinoma (HCC). This strategy utilizes red blood cells (RBCs) to deliver DNA vaccine-encapsulated polymeric nanoparticles to the spleen, enhancing antigen presentation and T-cell response activation (31). The study reported not only a halt in tumor progression but also, when combined with anti-PD-1 therapy, complete tumor regression and prevention of lung metastases. This dual approach induced a robust systemic immune response and long-term immunological memory, highlighting the potential of integrating targeted delivery with immune checkpoint inhibition. Another study focused on a synthetic multiepitope DNA vaccine, utilizing whole-exome sequencing and RNA-seq for precise neoantigen identification. The vaccine, encapsulated in a liposome and optimized for dendritic cell uptake, demonstrated significant efficacy in inhibiting melanoma growth and reducing lung metastasis in a mouse model (32). The vaccine promoted dense intratumoral infiltration of CD8^+^ T-cells, which effectively targeted melanoma cells, underscoring the vaccine’s capability to elicit a potent cellular immune response tailored to individual tumor profiles.

Furthermore, a novel DNA nanodevice has been developed for precise vaccine delivery, utilizing a sulfonium-driven mechanism for controlled antigen release. This technology ensures the stability and targeted delivery of neoantigens, enhancing cytokine secretion and promoting a strong CD8^+^ T-cell response (33). *In vivo* studies showed significant prevention of lung metastases and, in some cases, complete tumor regression, demonstrating the potential of DNA nanodevices as effective and precise modalities for tumor immunotherapy.

#### RNA-based vaccines

RNA-based neoantigen vaccines are at the forefront of advancing personalized cancer immunotherapy, utilizing specific tumor mutations to stimulate an immune response against cancer cells. RNA vaccines, which include messenger RNA (mRNA), circular RNA (circRNA), and self-amplifying RNA (saRNA) vaccines, are gaining traction for their ability to elicit a robust immune response without the need for live virus or protein production (37). The core concept of RNA vaccines is the delivery of genetic material—typically mRNA or other RNA forms—into cells, instructing them to produce tumor-associated antigens that are recognized by the immune system. This leads to both humoral and cellular immune responses, targeting the tumor for destruction. Unlike DNA vaccines, RNA vaccines do not require nuclear entry, which allows for a faster onset of antigen expression and immune activation (38). A novel strategy involving the noninvasive transdermal administration of mRNA vaccines encoding multivalent neoantigens has shown significant potential in inhibiting melanoma growth. In this approach, mRNA encoding three neoantigens was encapsulated into mannosylated chitosan-modified ethosomes for transcutaneous immunization (39). This vaccine format not only induced robust dendritic cell maturation but also significantly increased pro-inflammatory cytokines like TNF-alpha, IFN-gamma, and IL-12 in plasma and tumor tissues, leading to enhanced infiltration of CD4^+^ and CD8^+^ T cells into the tumor microenvironment. Importantly, when combined with siRNA targeting PDL1, the therapy exhibited a synergistic effect, suggesting the potential of combining mRNA vaccines with other immunomodulatory agents for enhanced therapeutic efficacy.

Another promising RNA vaccine platform is based on circular RNA (circRNA). Unlike linear mRNA, circRNAs are covalently closed RNA molecules, making them more stable and less prone to degradation. This stability enhances their ability to sustain protein expression, which is critical for maintaining persistent immune stimulation. One study explored a circRNA-based vaccine platform that effectively induced dendritic cell activation and subsequent T-cell-mediated tumor cell killing (40). By encapsulating neoantigen-encoded circRNAs within lipid nanoparticles, this platform showed significant tumor immunotherapy efficacy in murine models, highlighting the versatility of RNA structures in vaccine design and their potential for broader applications. Additionally, self-amplifying RNA (saRNA) vaccines are another advanced RNA vaccine technology. These vaccines incorporate RNA that encodes not only the tumor antigen but also viral replication machinery, enabling the amplification of the RNA once inside the cells. This results in higher antigen expression and a more potent immune response with lower required doses (41). saRNA vaccines have demonstrated promising results in preclinical studies and are being actively developed for cancer immunotherapy, offering a potential solution for enhancing vaccine efficacy with smaller doses.

Moreover, mRNA vaccines continue to show promise in cancer immunotherapy, as evidenced by a study that used RNA-seq data to identify tumor-specific neoantigens for mRNA vaccine development in hepatocellular carcinoma (HCC). By profiling tumor neoantigens and constructing immune clusters, researchers were able to identify which patient subgroups might benefit most from vaccination (2). Patients classified within certain immune clusters exhibited differential responses, underscoring the importance of personalized vaccine design based on detailed tumor and immune profiling to optimize therapeutic outcomes.

#### Dendritic cell vaccine

Dendritic cell (DC)-based vaccines represent a promising frontier in the field of personalized neoantigen-based cancer vaccines (Figure 4D). These vaccines harness the body’s own antigen-presenting cells to provoke a more robust and targeted immune response against cancer cells (42). The uniqueness of dendritic cell vaccines lies in their ability to be loaded with specific neoantigens identified from an individual’s tumor, enhancing the specificity of the immune response while maintaining a strong safety profile due to their precision. Recent advancements, as highlighted in the literature, have demonstrated that personalized dendritic-cell-based vaccines can be loaded with various antigens, including neoantigens, to elicit strong T-cell responses (43). These vaccines take advantage of the key role dendritic cells play as the primary antigen-presenting cells in the immune system, capable of activating not only T cells but also supporting the activation of B cells, thereby facilitating a comprehensive adaptive immune response.

Challenges remain in optimizing the delivery and efficacy of these vaccines. Issues such as ensuring consistent and targeted delivery of neoantigens to dendritic cells, overcoming the immunosuppressive tumor microenvironment, and the scalability of personalized vaccines are critical hurdles (42). Despite these challenges, the potential of dendritic cell vaccines to initiate strong and specific immune responses makes them a significant area of interest for the development of effective cancer treatments, particularly in the realm of personalized medicine (44). As research progresses, it is expected that these vaccines will play an increasingly central role in the treatment of cancers with high mutational burdens and diverse neoantigen landscapes.

#### Bispecific antibodies

Bispecific antibodies (BsAbs) are a novel class of therapeutic agents that can simultaneously bind two different epitopes, potentially enhancing the specificity and efficacy of immunotherapies, including neoantigen-based cancer vaccines (Figure 4A) (45). The studies on bispecific antibodies highlight their role in targeting mutant RAS neoantigens, which are common in various cancers and are traditionally challenging to target due to their intracellular nature. For instance, bispecific antibodies have been developed to specifically recognize peptide-HLA complexes derived from recurrent RAS mutations, such as G12V and Q61H/L/R, without cross-reacting with the wild-type form (46). This specificity facilitates the precise elimination of cancer cells presenting these mutations by activating T cells and directing them to kill targeted cancer cells. Moreover, the advancement of bispecific antibodies targeting CD40 and tumor-associated antigens shows promise in enhancing antigen presentation and T-cell priming, which are crucial for effective antitumor responses (47). This strategy has demonstrated superior antitumor activity compared to monospecific antibodies by promoting cross-priming of T cells and potentially remodelling the tumor microenvironment to be more immunogenic.

The ongoing development and refinement of bispecific antibodies, including their ability to target intracellular antigens through novel delivery systems, present a versatile platform for cancer immunotherapy. However, challenges such as immune escape, T cell exhaustion, and the need for highly specific targeting to avoid off-target effects continue to be significant hurdles in the clinical application of bispecific antibodies in neoantigen-based therapies (48). The integration of these innovative approaches could significantly enhance the precision and personalization of cancer immunotherapies, heralding a new era in the management of malignancies with neoantigen-specific strategies.

#### Genetically engineered anti-tumor immune cells

Genetically engineered anti-tumor immune cells, such as chimeric antigen receptor T-cells (CAR-T) and T cell receptor-engineered T-cells (TCR-T), represent a significant advancement in personalized neoantigen-based cancer vaccines (Figures 4B, C) (49). These therapies are designed to enhance the immune system’s ability to recognize and destroy cancer cells by targeting neoantigens that arise from tumor-specific mutations (50). The power of CAR-T and TCR-T lies in their ability to be customized to recognize unique antigens present on an individual’s cancer cells, making them highly effective against tumors that express these specific neoantigens. Recent studies have demonstrated the potential of these therapies in achieving significant clinical responses, particularly in hematological malignancies and some solid tumors (51). CAR-T cells have been engineered to target specific cancer cell surface antigens, while TCR-T therapies are tailored to recognize intracellular antigens presented by major histocompatibility complex (MHC) molecules (52). This approach allows for the targeting of a broader range of cancer-specific mutations compared to traditional therapies.

Despite their promise, several challenges remain in the widespread application of these therapies. The identification and validation of neoantigens that can be targeted safely and effectively is complex and requires extensive bioinformatic analysis and validation (53). Additionally, the manufacturing of these personalized therapies is technically demanding, expensive, and time-consuming. Moreover, managing the severe immune-related adverse effects, such as cytokine release syndrome and neurotoxicity, poses significant clinical management challenges (33). Overall, genetically engineered anti-tumor immune cells offer a promising but challenging frontier in cancer treatment, representing a profound shift towards more personalized and effective immunotherapy strategies (49). As research progresses, optimizing the selection of target neoantigens, improving manufacturing processes, and better managing side effects are critical for maximizing the therapeutic potential and accessibility of CAR-T and TCR-T therapies.

### Delivery system

The delivery of these vaccines is optimized through several platforms, such as lipid nanoparticles and dendritic cell vaccines (8). These delivery methods are critical for the effective administration of the vaccine components, ensuring that they reach the target cells and tissues. Lipid nanoparticles, for example, help stabilize RNA vaccines and enhance their delivery into cells, while dendritic cell vaccines involve programming dendritic cells outside the body to present neoantigens directly to the immune system, thereby initiating a targeted immune response (54). One particularly promising approach has been the development of a mucosal vaccine delivery vehicle using Lactococcus lactis to secrete mutant KRAS neoantigens targeting colorectal cancer. This system utilizes a novel signal peptide that optimizes secretion efficiency and antigen presentation, crucial for inducing a robust mucosal immune response (55). The ability of this vehicle to selectively target the gastrointestinal tract and induce specific IgA responses without causing systemic side effects illustrates the potential of site-specific delivery systems for cancer vaccines. Additionally, the use of advanced biomaterials to enhance delivery efficiency is gaining traction. For example, a study involving the delivery of spike nanoparticle neoantigen vaccines for hepatocellular carcinoma utilized virus-like silicon vaccine particles to co-deliver neoantigens and adjuvants directly to dendritic cells (56). This method leverages caveolin-mediated endocytosis to bypass cellular barriers and enhance lymph node drainage, significantly improving T-cell activation and tumor infiltration, which is critical for robust antitumor immunity. Moreover, the use of thiolated nano-vaccines has shown significant promise in delivering neoantigens directly to the cytosol, avoiding degradation pathways that typically limit vaccine efficacy (9). This strategy enhances the local concentration of antigens and adjuvants, promoting more effective dendritic cell activation and T-cell responses. When combined with immune checkpoint blockade, such vaccines have achieved remarkable tumor control, illustrating the potential for integrating vaccine delivery with other therapeutic modalities to enhance overall cancer treatment outcomes.

### Clinical trials and evidence

Drawing from the evolving landscape of personalized neoantigen-based cancer vaccines, recent clinical trials offer insights into their therapeutic potential and associated challenges across various cancer types. One pivotal study, a phase I trial, investigated the use of adjuvant autogene cevumeran, a personalized RNA neoantigen vaccine, in patients with pancreatic ductal adenocarcinoma (PDAC). This trial combined the vaccine with the immune checkpoint inhibitor atezolizumab and a potent chemotherapy regimen, mFOLFIRINOX (38). The vaccine was found to induce neoantigen-specific T cell responses in 50% of the treated patients, and these responses were associated with a notable increase in recurrence-free survival; patients with T cell responses showed a median recurrence-free survival that was not reached, compared to 13.4 months in non-responders, indicating a substantial therapeutic benefit. In hepatocellular carcinoma (HCC), a phase 1/2 trial tested a DNA plasmid-based personalized therapeutic cancer vaccine (PTCV) coadministered with pembrolizumab and interleukin-12. The study aimed to assess both safety and immunogenicity, with a focus on treatment efficacy. The trial reported an objective response rate of 30.6%, with 8.3% of patients achieving a complete response (57). Notably, neoantigen-specific T cell responses were confirmed in 86.4% of evaluable patients, highlighting the strong immunogenic potential of the vaccine. Another significant study, a phase 2b trial, evaluated the combination of mRNA-4157 (V940), an individualized neoantigen therapy, with pembrolizumab versus pembrolizumab alone in patients with resected high-risk melanoma. The combination aimed to enhance recurrence-free survival (58). Preliminary results suggested that the addition of the neoantigen vaccine to pembrolizumab could improve clinical outcomes, showing a lower hazard ratio for recurrence or death of 0.561, which approached statistical significance with a p-value of 0.053.

Complementing these findings, a phase I trial in non-small cell lung cancer (NSCLC) demonstrated that a dendritic cell vaccine was administrable to 60% of participants, with 83% exhibiting systemic T cell responses. However, 50% of these patients experienced disease recurrence within two years, illustrating the challenges in achieving sustained disease control (59). Follicular lymphoma was addressed in a trial using synthetic long peptide vaccines, derived from whole-exome and RNA sequencing, which successfully treated all enrolled patients clinical trials data (60). Metastatic melanoma patients in a phase Ib trial showed strong immunogenic responses and a robust safety profile with the NOUS-PEV vaccine combined with pembrolizumab clinical trials data (61). A phase 1 trial in triple-negative breast cancer highlighted an 87.5% recurrence-free survival rate at 36 months, with 78% of patients developing specific immune responses following treatment with a neoantigen DNA vaccine clinical trials data (62). Moreover, a pilot study for metastatic soft tissue sarcoma combining LTX-315 with adoptive cell therapy demonstrated stable disease up to 208 days in some patients, with 50% showing *de novo* T-cell responses against predicted neoantigens clinical trials data (63). These trials collectively highlight the nuanced potential of neoantigen-based vaccines in oncology, showcasing their ability to elicit significant immune responses and manage safety profiles across diverse cancer types, albeit with variable therapeutic efficacy that underscores the necessity for continued optimization.

## Challenges and limitations

**FIGURE 5:**
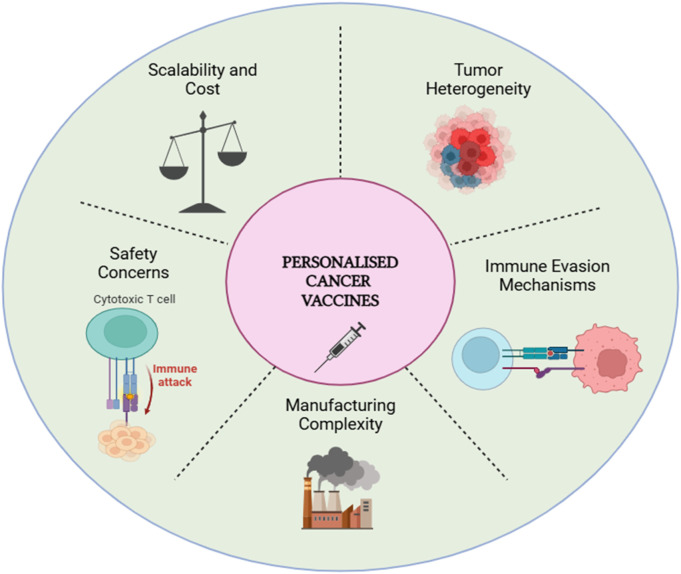
Challenges in developing personalized cancer vaccines.

The development and clinical deployment of personalized neoantigen-based cancer vaccines face a myriad of challenges, chief among them tumor heterogeneity, immune evasion, manufacturing complexity, safety concerns, and the limited response rate in certain patient populations (Figure 5) (4). Tumor heterogeneity, encompassing both genetic and phenotypic diversity, complicates the precise identification and targeting of neoantigens. Mutations within a single tumor can vary widely, necessitating highly customized vaccine formulations (59). This inherent variability in tumor biology often leads to differential vaccine efficacy, emphasizing the need for adaptable treatment strategies that can accommodate this complexity. Moreover, tumors frequently deploy multiple immune evasion strategies, such as the expression of checkpoint proteins (e.g., PD-L1) that dampen immune responses, and the modulation of the tumor microenvironment to inhibit T-cell infiltration and activity (60). These mechanisms not only reduce the immunogenicity of neoantigens but also hinder the predictability of vaccine responses, posing significant challenges to the robustness and universality of these therapeutic interventions.

On the manufacturing front, the bespoke nature of these vaccines introduces significant logistical and financial hurdles. Each vaccine must be individually tailored and manufactured based on the patient’s tumor-specific neoantigens, a process that is resource-intensive and time-consuming, potentially delaying treatment for patients with aggressive cancers (61). Additionally, scalability remains a major concern, with production costs and technical challenges limiting the widespread clinical application of personalized therapies. The need for ultra-sensitive and precise genomic and proteomic analyses further adds to the complexity, necessitating specialized infrastructure that may not be available in resource-limited settings. Safety concerns are also critical, as the potent stimulation of the immune system by neoantigen vaccines can lead to severe adverse effects, including autoimmune reactions and off-target effects (62). These may manifest in various forms, such as cytokine storms, where the excessive release of pro-inflammatory cytokines can result in systemic inflammation and organ damage, neuropathy, where nerve damage from an overactive immune response leads to debilitating symptoms, and multiple organ failure in extreme cases (64). Additionally, neurological side effects have been observed in patients undergoing immunotherapy, with immune-related adverse events potentially exacerbating pre-existing conditions or leading to new neurological impairments (65). These safety issues necessitate rigorous patient monitoring, early detection systems, and the development of strategies to mitigate such risks, which complicates the clinical implementation of these vaccines. Furthermore, limited response rates are observed in certain patients, with some individuals showing little to no immune response despite receiving personalized vaccines, likely due to tumor-induced immune suppression or insufficient immune priming. This variability in response underscores the importance of identifying biomarkers that can predict the likelihood of a positive response to neoantigen vaccination, and adjusting treatment protocols accordingly.

Ethical considerations also play a critical role in the deployment of personalized cancer vaccines, particularly in terms of equitable access, informed consent, and the potential long-term effects of genetic modifications on patients. Each of these challenges highlights the complexity of personalized neoantigen-based cancer vaccines. To improve their efficacy, safety, and widespread applicability, further research is required to understand the underlying mechanisms of action, refine manufacturing processes, and develop innovative solutions for patient-specific treatment plans. Continued efforts in overcoming these barriers are essential to realizing the potential of personalized cancer vaccines as a mainstream therapeutic option.

## Future perspectives and directions

As the field of personalized neoantigen-based cancer vaccines continues to evolve, several promising future directions are emerging that may significantly enhance their clinical application and effectiveness. One of the most exciting prospects lies in the continued integration of advanced sequencing technologies with computational tools to improve the identification and selection of neoantigens. The use of next-generation sequencing (NGS) and bioinformatics tools enables the detection of a broad spectrum of mutations across a patient’s tumor, paving the way for the creation of personalized vaccines that target the specific neoantigens derived from these mutations (66). This refinement in neoantigen identification is expected to lead to more precise vaccines, improving patient outcomes by enhancing the specificity and immunogenicity of the therapeutic targets.

Moreover, combination therapies are increasingly seen as a critical strategy for improving the overall effectiveness of personalized cancer vaccines. Combining neoantigen vaccines with immune checkpoint inhibitors (ICIs) or adoptive T-cell therapies, such as CAR-T cells, has demonstrated the potential to overcome immune suppression within the tumor microenvironment, which can otherwise hinder vaccine efficacy (67). For example, combining personalized vaccines with PD-1 inhibitors has already shown enhanced immune responses and improved clinical outcomes in several cancer types, including melanoma and non-small cell lung cancer (NSCLC) (57). This combination approach not only boosts the immune system’s ability to recognize and attack tumor cells but also helps mitigate the mechanisms by which tumors evade immune surveillance.

The development of more effective vaccine delivery systems is another area of significant innovation. Nanoparticle-based delivery systems, such as those utilizing PLGA-PEI nanoparticles, are gaining traction for their ability to enhance the stability and immune activation potential of neoantigen vaccines. These systems can encapsulate antigens and adjuvants in a way that allows for more efficient targeting of dendritic cells, which are critical for initiating a robust T-cell-mediated immune response (68). Moreover, these nanoparticle-based platforms can provide controlled release of the vaccine components, improving both efficacy and safety by reducing the risk of adverse reactions.

Another promising area is the incorporation of real-time monitoring and adaptive clinical trial designs to accelerate the evaluation of personalized vaccines. As tumor heterogeneity remains a major challenge in the field, adaptive trial designs that incorporate real-world data and continuous monitoring of patient responses can allow for more flexible and efficient assessment of vaccine effectiveness (69). By utilizing data from diverse patient populations and tumor types, these adaptive trials can quickly identify which neoantigens and treatment combinations are most effective, helping to expedite the regulatory approval process.

## Conclusion

Personalized neoantigen-based cancer vaccines represent a significant advancement in the field of oncology, offering a promising approach to cancer treatment that leverages the unique genetic profile of an individual’s tumor. By targeting specific neoantigens, these vaccines aim to elicit robust immune responses that are precisely directed against tumor cells, potentially improving therapeutic outcomes while minimizing side effects. However, challenges remain in accurately identifying immunogenic neoantigens and developing effective delivery systems to ensure the optimal presentation of these antigens to the immune system. In light of recent clinical trial data, the feasibility of clinical adoption is improving, with several trials demonstrating promising efficacy and immune responses across various cancer types, though challenges remain in achieving sustained disease control. Continued advancements in neoantigen identification, combination therapies, and delivery systems will be essential to maximize the therapeutic potential and clinical adoption of these vaccines. As research progresses, these vaccines are poised to fundamentally alter the landscape of cancer treatment, making it more personalized and effective.
